# Barriers to providing quality emergency obstetric care in Addis Ababa, Ethiopia: Healthcare providers’ perspectives on training, referrals and supervision, a mixed methods study

**DOI:** 10.1186/s12884-015-0493-4

**Published:** 2015-03-29

**Authors:** Anne Austin, Hanna Gulema, Maria Belizan, Daniela S Colaci, Tamil Kendall, Mahlet Tebeka, Mengistu Hailemariam, Delayehu Bekele, Lia Tadesse, Yemane Berhane, Ana Langer

**Affiliations:** Maternal Health Task Force, Women and Health Initiative, Harvard T. H. Chan School of Public Health, Boston, USA; Addis Continental Institute of Public Health, Addis Ababa, Ethiopia; Institute for Clinical Effectiveness and Health Policy, Buenos Aires, Argentina; Federal Ministry of Health, Addis Ababa, Ethiopia; St Paul Hospital Millennium Medical College, Addis Ababa, Ethiopia

**Keywords:** Quality of care, Obstetric emergencies, Training, Supportive supervision, Urban referrals, Maternal health, Ethiopia, Healthcare provider perspectives

## Abstract

**Background:**

Increasing women’s access to and use of facilities for childbirth is a critical national strategy to improve maternal health outcomes in Ethiopia; however coverage alone is not enough as the quality of emergency obstetric services affects maternal mortality and morbidity. Addis Ababa has a much higher proportion of facility-based births (82%) than the national average (11%), but timely provision of quality emergency obstetric care remains a significant challenge for reducing maternal mortality and improving maternal health. The purpose of this study was to assess barriers to the provision of emergency obstetric care in Addis Ababa from the perspective of healthcare providers by analyzing three factors: implementation of national referral guidelines, staff training, and staff supervision.

**Methods:**

A mixed methods approach was used to assess barriers to quality emergency obstetric care. Qualitative analyses included twenty-nine, semi-structured, key informant interviews with providers from an urban referral network consisting of a hospital and seven health centers. Quantitative survey data were collected from 111 providers, 80% (111/138) of those providing maternal health services in the same referral network.

**Results:**

Respondents identified a lack of transportation and communication infrastructure, overcrowding at the referral hospital, insufficient pre-service and in-service training, and absence of supportive supervision as key barriers to provision of quality emergency obstetric care.

**Conclusions:**

Dedicated transportation and communication infrastructure, improvements in pre-service and in-service training, and supportive supervision are needed to maximize the effective use of existing human resources and infrastructure, thus increasing access to and the provision of timely, high quality emergency obstetric care in Addis Ababa, Ethiopia.

**Electronic supplementary material:**

The online version of this article (doi:10.1186/s12884-015-0493-4) contains supplementary material, which is available to authorized users.

## Background

In 2006, the Ethiopian Federal Ministry of Health (FMOH) developed the National Reproductive Health Strategy (2006–2015) that focuses specifically on improving facility infrastructure, training health care providers and promoting referrals to health facilities for birth [1]. Despite on-going efforts to implement these strategies, the maternal mortality ratio (MMR) has remained high. The 2011 Ethiopian Demographic and Health Survey (EDHS) estimated a MMR of 676 maternal deaths per 100,000 live births, almost no change from the 2005 EDHS estimates of 673 [2]. Currently, UNFPA estimates that 22,000 Ethiopian women and girls die annually as a result of pregnancy and childbirth complications and an additional 500,000 suffer from pregnancy-related morbidities [3]. While the reasons for high maternal mortality ratios in Ethiopia are multi-factorial [4-6], low rates of facility-based deliveries, lack of trained personnel and emergency obstetric services at facilities, and inefficient referral systems for obstetric emergencies are key health system weaknesses [1,4-6]. Access to and utilization of facility based maternal health services is more common in Addis Ababa than other parts of Ethiopia. According to the 2011 EDHS 81.9% of women in Addis Ababa reported giving birth in a facility and 58% reported that a medical doctor attended their birth. In comparison, national rates for facility-based births were 11% and only 3.6% of births were attended by a physician [2]. Although infrastructure, facility readiness and provider capacity indicators in Addis are better than those of more remote regions, indicators used to assess Emergency Obstetric and Newborn Care (EmONC) indicate that the situation in Addis is worse than in other major cities, such as Harar and Dire Dawa, and in some cases worse than national averages [4]. For example, in Addis Ababa there are 21hospital beds available per 1000 births as compared to the national average of 32 beds per 1,000 births [4]. In this urban context, improving the quality of emergency obstetric care is crucial for maternal health.

In 2012, the Federal Ministry of Health (FMOH) and the Addis Ababa Regional Health Bureau (AARHB) developed an operational manual for a regional Emergency Obstetrics Referral Network in Addis Ababa and established eight health center to hospital referral networks in the city [7]. The urban referral networks aim to optimize the use of scarce healthcare resources by ensuring that care is delivered at the appropriate level of facility and to reduce maternal mortality and morbidity associated with delays in reaching and receiving appropriate care [8].

To evaluate the progress of these country-led efforts to improve the availability and quality of emergency obstetric care, we conducted a mixed methods study with healthcare providers working in one of the eight emergency obstetric referral networks in Addis Ababa to assess the implementation of national referral guidelines, training, and staff supervision as they pertain to the provision of emergency obstetric care. The functioning of the referral network, training, and supervision were prioritized for evaluation because they are central to the FMOH’s priorities for improving maternal health, have been shown to improve the quality of maternal health services, can be implemented in the short-to-medium term, and maximize the use of existing health system resources [1,7,9].

Studies have noted that the engagement of providers in the identification and evaluation of interventions to improve the quality of emergency obstetric care are invaluable [9,10]. Provider perspectives are also needed to identify high-impact interventions that address poor quality emergency obstetric services [9-13]. A recent study in rural Ethiopia found that providers had relevant insights into the factors that lead women to seek facility based births [10]. As urban care seeking profiles in Addis Ababa are markedly different from rural care seeking profiles, the knowledge and perceptions of urban maternal health providers can contribute to identifying barriers to quality emergency obstetric care in Addis Ababa.

In this paper, we describe and discuss the findings of a baseline study that identified barriers to the provision of quality emergency obstetric care, in order to develop interventions.

## Methods

A census, in March 2013, of the maternal health providers in the St. Paul’s Hospital Millennium Medical College (SPMMC) hospital- health center network, identified 138 maternal health providers. The sample for the quantitative survey consisted of 111 of 138 (80%) of the healthcare workers providing maternal health services (antenatal (ANC), post natal, and delivery care) in the referral network. Staff providing these three services often rotate between the ANC, post natal and delivery wards, so providers from all three levels of care were included in the survey. Three providers declined to complete the questionnaire, a refusal rate of 2% (3/111). Twenty-four providers (17%, 24/111) were not available to complete the survey because of annual leave, off site training or being off duty. The data collection tools were piloted and pretested in a comparable hospital-health center network in Addis Ababa: the Ghandi Memorial Hospital and the Kirkos health center. The quantitative data collection instrument can be found in Additional file 1. Data were collected by six trained data collectors at the health facilities.

The key informant interview participants were purposively selected from the population of maternal healthcare providers in the referral network based on their professional responsibilities. The participants for the in-depth interviews included the medical directors from each of the networked health centers, the liaison officers who are responsible for maternal referrals, maternal and child health coordinators, and senior nurses and midwives from the labor rooms. The structured interview guide that was used can be found in Additional file 2. Of the 31 respondents identified, two were unavailable to participate, and none refused to participate. This resulted in a sample size of 29 with three participants from the hospital and 26 from health centers. Each respondent was interviewed individually at his/her place of work by a team of trained data collectors from the Addis Continental Institute of Public Health (ACIPH): one interviewer and a note taker. Both data collectors were trained in the use of the interview guide and qualitative methodologies before data collection began. Interviews were conducted in private offices, and ranged in duration from 22to 74 minutes. Interviews were audio recorded, transcribed in Amharic, and then translated to English [11]. During the coding process, translations from Amharic were back-translated and validated within the context of the interview by a native Amharic speaker (HG); no discrepancies from the original translation to English were identified.

Qualitative and quantitative data was collected in March of 2013. Data collectors and study participants were aware that the goal of the study was to assess factors (including provider knowledge and confidence in their skills and facility resources) that influence the provision of emergency obstetric services in order to develop interventions to improve the quality of care.

In-country ethical clearance was obtained from the ACIPH Institutional Ethical Review Board. Informed consent was obtained from all study participants. The study is registered with the National Institutes of Health in the United States of America: NCT01802957.

### Analysis

*A priori* themes were coded based on the study objectives and emergent themes were identified based on the narratives of research participants. Interviews were analyzed and coded by one researcher (AA) and the coding was verified by a second researcher (MB). This analysis draws on participating providers’ responses to queries about three main issues: the functioning of the newly developed referral system; current staff capacity and the need for technical training to appropriately handle obstetric emergencies; and status of and satisfaction with the supervisory visits. The quantitative survey provides complementary information about 1) healthcare provider characteristics 2) receipt of and satisfaction with in-service training 3) perception of supervision and job satisfaction. Data were double entered using Epi Info software version 3.5.1 and descriptive statistics were generated using SAS V9.2. When comparing and contrasting the qualitative and quantitative findings during the interpretation, we adapted the classic “three delays” model that has been developed by Thaddeus and Maine and fruitfully applied by other researchers, to conceptualize the barriers to quality obstetric care identified by providers in Addis Ababa, Ethiopia [8,12-16].

## Results

### Study population

The final sample of 29 individuals who completed the qualitative study (i.e., in-depth interviews) included the Medical Directors, the Directors of Maternal Child Health services, nurses and midwives who work in the labor wards, and the person responsible for coordinating referrals (referral focal person). Fifteen of the qualitative study respondents were female (52%, 15/29), and the respondents’ average age was 29. The survey respondents (n = 111) were predominantly nurses (49%, 54/111) and midwives (40%, 44/111), with a smaller number of health officers (5%, 6/111), physicians (3%, 3/111), and medical interns (3%, 3/111).72% (80/111) of the surveyed providers were female; the average age of providers surveyed was also 29 years.

#### Barriers to providing quality emergency obstetric care

**Figure 1:**
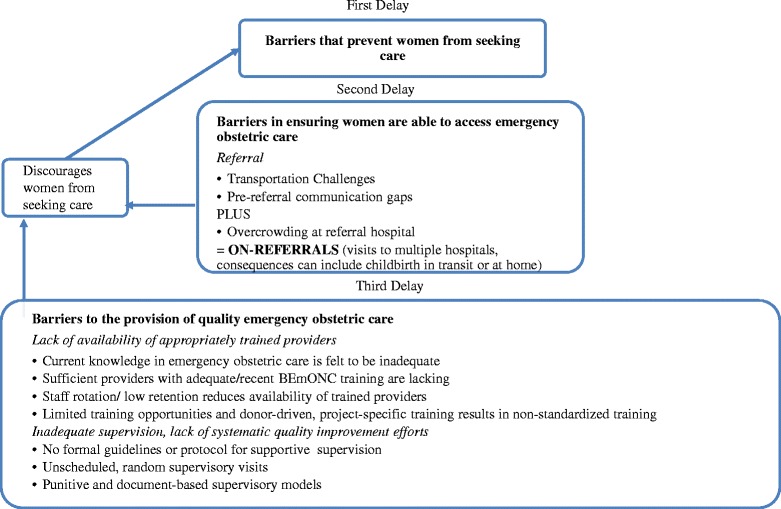
**Delay phases and barriers to the provision of quality emergency obstetric care, as identified by healthcare providers in Addis Ababa, Ethiopia (adapted from Thaddeus & Maine, 1994).**

Figure 1 summarizes how the identified barriers exacerbate the second delay (reaching appropriate care), third delay (provision of appropriate care), as well as how they may contribute to the first delay (seeking care) if women lose confidence that they will receive needed obstetric services in a timely fashion by attending a facility.

Healthcare providers identified lack of transportation and communication infrastructure and overcrowding at the referral hospital as challenges for the smooth functioning of the referral network, as well as insufficient pre-service and in-service training in obstetric emergencies, and lack of supportive supervision as barriers to the provision of timely, quality emergency obstetric care.

### Transportation and communication are barriers to referral network functioning

The referral guidelines developed by the FMOH and the AARHB were clearly understood by respondents interviewed at the health center level. Respondents knew that SPMMC is their referral hospital for obstetric emergencies, and had clear algorithms for documenting the out- referral process. Universally, health center providers reported filling out referral slips. Nevertheless, limited availability of transportation and lack of communication and coordination between the health centers and the hospital before and after referrals were identified as hindering the efficiency of the referral system.

Challenges in securing transportation complicate referrals and contribute to delays in providing emergency obstetric services. Most of the health centers said that the average travel time to the hospital was less than an hour, but noted that the ambulance system was unreliable due to driver shortages and vehicle breakdowns.

*“In the day time, there is no ambulance available from the sub city. It may be due to driver shortages, or the fact that the ambulance is not working.”*(Female, Health Center, BSC Nurse).

When ambulances are not available, referred women have to make their own way to the hospital. Several respondents noted occasions when women in labor had to walk, take a taxi or find another means of transportation to the hospital.

Additionally, when women are referred in an ambulance, referring facilities are mandated to send a provider to accompany the woman. While respondents agreed with this requirement in principle, they noted that it strained facility capacity, particularly in the evening when only one or two providers are on duty at the health center level. Command post ambulances have a dedicated provider that travels with them, and respondents said that this makes these ambulances a preferred means of transportation because “*there is no need for facility staff to accompany the woman (when the command post ambulance system is used). If we use a regular ambulance, one of our staff members must accompany the mother.”*(Male, Health Center, Health Officer).

### Communication gaps between facilities

Guidelines state that “Obstetric referrals will be mediated by communication between referring health facility and receiving health facility through the liaison officer/referral coordinator of both facilities” [7]. Respondents knew that they should call the hospital before referring a woman and recognized the importance of doing so, particularly in order to assess whether the hospital could receive the woman. For instance a health center health officer stated that *“One thing that we could improve upon is ongoing phone contacts with the liaison office. The liaison office is required to check for the presence of a bed, and if (SPMMC) is willing to accept the referral.”*(Male, Health Center, Health Officer). However, lack of dedicated phone lines hampered pre-referral communication. Providers explained that “*the phone is not always accessible, and women are sometimes referred without a call”* (Male, Health Center, Health Officer). Despite awareness, lack of dedicated communication infrastructure was a barrier to implementing the referral protocol.

*“In theory, we are supposed to call St. Paul hospital to check on the availability of beds, but this is not applied practically. We are not provided with phones for communication. In practice, we call the command post (ambulance), and the command post (ambulance) takes the mother to St. Paul*.” (Female, Health Center, BSC Nurse)

Lack of pre-referral communication and high-patient volume at SPMMC resulted in women being referred to other facilities after they arrived at SPMMC.

*“We accept referrals if there is a bed, but if there is no bed, we call other hospitals so that the mother can be referred to a facility where there are available beds”* (Female, Hospital, BSC Midwife).

Respondents recognized the consequent delay in the provision of appropriate emergency obstetric care as a threat to women’s health, and described cases in which on-referral resulted in women giving birth at home or in transit.

*“When mothers are referred to the hospital, the hospital fails to accept the referrals. The mothers spend the whole night searching for a hospital that is willing to accept or admit her. She carries the referral paper from hospital to hospital. Nobody knows what happens to those mothers, whether they are alive or dead.”* (Male, Health Center, BSC Nurse)

*“One of our mothers was sent to three hospitals and got exhausted at the fourth hospital. She gave birth to her child on the way home. Women go everywhere searching for a hospital, and end up having a home delivery.”* (Male, Health Center, BSC Nurse)

The scenario of women being referred from hospital to hospital because of lack of beds and inadequate communication between the different levels of the health system was mentioned by respondents across facilities, and among all cadres of provider. Respondents also shared experiences suggesting that on-referral is a disincentive to facility-based childbirth. A nurse from a health center clearly articulated this phenomenon, explaining:

“*A mother in our catchment area recently delivered at home. When we asked her why, she replied that when they come to the health center, and are in need of referral, they may be referred to a hospital; the hospital may not accept them for proper management; because there may be no beds available. So it is better for them to stay and deliver at home.”* (Female, Health Center, BSC Nurse)

To alleviate overcrowding at the referral hospital, several respondents suggested that low-risk women who are referred or present spontaneously at SPMMC should be referred to lower level health centers. At the time of the interviews none of the facilities had implemented this system.

### Training gaps in BEmONC and CEmONC

Lack of provider training in Basic Emergency Obstetric and Newborn Care (BEmONC) and Comprehensive Emergency Obstetric and Newborn Care (CEmONC) compromises health facilities ability to provide the needed services at the appropriate facility level. Only 10% (4/42) of providers at SPMMC and 25% (17/69) of the providers at the health center level reported having completed one or more components of BEmONC training during the twelve months preceding the survey; none reported in-service training on all components of BEmONC. Among medical post-graduate interns and doctors at SPMMC only 12% (5/42) had received at least one component of in-service CEmONC in-service training and none had completed all components of CEmONC training.

During in-depth interviews, healthcare providers stated that their institutions lacked sufficient trained personnel to respond to obstetric emergencies and identified weaknesses in both pre-service and in-service training. Recurrent themes included inadequate attention to obstetric emergencies in pre-service training, the need for regular in-service training in obstetric emergencies to update knowledge, and that available trainings and mechanisms for selecting providers for training opportunities are not standardized.

BEmONC was not a standard part of pre-service training for all maternal healthcare providers. Respondents stated that the lack of pre-service training left their healthcare facilities unprepared to identify and respond to obstetric emergencies.

*We have six midwives here, but no one is trained in BEmONC. Six midwives are available but we cannot detect complicated issues. So having six midwives is just a number—what is the relevance of having six midwives?* (Male, Health Center, BSC Nurse).

Other providers had been trained to manage obstetric emergencies as part of their pre-service degree program but expressed the need for refresher trainings to ensure the retention of skills, provider confidence, and that the most recent standard of care guidelines are being used.

*“So much has changed since we were first trained. There is no refresher course to ensure we are up to date on the best standards of care.”* (Female, Health Center, BSC Midwife)

Universally, the respondents showed very strong support for in-service training to handle obstetric emergencies. Respondents felt that in-service BEmONC training would improve facilities’ ability to appropriately manage obstetric emergencies. An illustrative comment was made by a nurse who opined that:

*“to provide good service, we need to improve our knowledge. We might be creating problems for our clients because we don’t have updated information or adequate knowledge.”* (Male, Health Center, BSC Nurse)

However, the current system of training described by healthcare providers neither privileged training in obstetric emergencies nor systematically selected the most appropriate providers to participate in training when it was offered. Healthcare providers reported that most training opportunities depend on external funding.

*“Trainings are done when there is a financial sponsor available. Most of the trainings do not come from the Addis Ababa Regional Health Bureau, but are collaborations between NGO’s and the Government”* (Male, Health Center, Referral Focal Person).

Consequently, opportunities for and implementation of trainings is not consistent between facilities or different cadres of healthcare providers and trainings tend to be sponsor-driven and project-specific. Perhaps as a consequence, 19 out of 40 (48%) of the survey respondents who reported receiving in-service training over the past year were trained in Prevention of Mother to Child Transmission of HIV, an area for which there is extensive external funding. Overall, only 26 of the 111 (24%) of providers who responded to the survey said they were satisfied with in-service training opportunities.

While some respondents reported that job functions or duration of service were taken into account when deciding who would attend training, the most common selection mechanism reported by respondents was a lottery system. Several health center directors described implementing an informal system of peer-to-peer training to increase the benefits and reach of scarce training opportunities.

*“We would be happy if every staff member could be trained. When staff members are trained, we arrange an orientation program when they come back, so that staff who were unable to take the training, can learn from them.”* (Male, Health Center, Health Officer).

Finally, relatively short duration of service and staff rotation reduced the benefit of in-service BEmONC training to individual healthcare institutions and in some cases, for the healthcare system as a whole

*“After receiving BEmONC training, some of those trained changed their field, and others resigned from this facility.”* (Male, Health Center, BSC Nurse*).*

The survey of providers found that 40% (44/111) had been at their current facility for less than a year and that 66% (73/111) of providers had been at their facility for less than two years.

### Barriers to ensuring job satisfaction and implementing supportive supervision efforts

In addition to exploring the functioning of the referral system and provider training in BEmoNC and CEmONC as factors that directly impact access to and availability of quality emergency obstetric care, we also assessed providers job satisfaction and experiences of supervision, as some research has shown that provider satisfaction and supervisory models have important consequences for retention of healthcare workers and affects quality of care [17,18].

In response to the quantitative survey, 83% (92/111) of providers reported that they were satisfied with the freedom they have to make important decisions about patient care independently, and 95% (105/111) were satisfied with the collaboration and team work between different cadres of health workers at their facility. On the other hand, only 50% (56/111) reported being satisfied with the supervision they received at their facility.

Qualitative analysis of interviews suggests that routine supervision tends to be traditional rather than supportive. Key characteristics that differentiate supportive supervision from traditional supervision are: involvement of staff members and a wider range of colleagues in evaluation; continuous supervision occurring in a variety of contexts rather than only periodic visits by external supervisors; provision of on-site technical support/training and joint problem solving instead of limited to reactive problem solving or directions from the supervisor; actions and decisions are recorded and followed-up as compared to no or irregular follow-up [19]. Respondents’ descriptions of irregular or unscheduled visits by external supervisors that were focused on record-keeping, attendance, and “fault finding” indicates that a traditional supervisory model persists in these health facilities.

*“Supervision visits are more of a fault finding. It is not supportive and discourages us due to the actions of the supervision teams.”*(Female, Health Center, Health Officer)

*“Existing programs only review the quality of your documentation, or whether you are present or absent. I don’t see anything that they are doing in order to facilitate my work. They are only interested in whether or not we are present.”* (Female, Hospital, BSC Midwife)

*“Most of the time, no one asks us about our problems during supervision, no one hears about our problems. What they want to do is copy already written and available data for the sake of reporting.” (*Male, Health Center, BSC Nurse)

*“In the future, it would be better to give support rather than supervising all the time. Feedback should be given to the health center about the problems and issues identified during the supervision, so that the health center can intervene. If the problem is beyond the health center, the gap should be filled by the supervising body.”* (Male, Health Center, BSC Nurse)

At the health center level, respondents viewed supervisory visits as an opportunity for the health center to highlight shortages of medical supplies, and to solicit the procurement of necessary goods. Some respondents expressed frustration that supervisory visits from the AARHB did not result in provision of needed supplies.

*“The AARHB comes to supervise us every three months. We do not have a vacuum, or suction machine. I commented on this when they came for supervision, but they did not fill our gap.”(*Female, Health Center, BSC Nurse)

Some respondents had experienced supportive supervision; however, their descriptions suggest that many of these programs, like many of the trainings, are donor and project specific.

Respondents who had participated in supportive supervision indicated that it was useful and effective for improving quality of care.

*“When we are provided with written feedback, it motivates us and helps us prepare action plans. This provides us with actionable projects. We have seen many changes because of the findings and feedback from supportive supervision visits.”*(Male, Health Center, Health Officer)

In some cases the respondents contrasted their positive experiences of supportive supervision from external agencies with their experiences of supervision from the AARHB.

*“The CDC implemented supportive supervision, and provided us with feedback… this (feedback) is not usual when the visit is undertaken by the AARHB”* (Female, Health Center, Health Officer)

During in-depth interviews, none of the respondents reported having received supportive supervision focused on emergency obstetric procedures or preparedness.

Overall, respondents expressed strong interest in supportive supervision and hope that such a supervisory system would provide constructive feedback, actionable quality improvement work plans and improvements in availability of needed medical supplies. Although the provision of supportive supervision falls under the mandate of the AARHB, there are currently no formal guidelines for the implementation of supportive supervision at the health center level.

## Discussion

Existing literature identifies various determinants for the three delays for preventive and emergency obstetric care. Major barriers to care identified in similar low and middle-income settings include sociocultural factors, perceived benefit/need, economic accessibility and physical accessibility of services [20,21]. Sociocultural factors, such as maternal age and education, and perceived need for care, are determinants for the ‘first delay,’ which influence the decision-making process regarding whether the mother seeks care. Unlike other areas of Ethiopia, in Addis Ababa 94% of women attend prenatal care and 82% seek facility births, suggesting that the first delay is not the most important barrier for reducing maternal mortality in this urban context, and that seeking facility based maternity services has become the norm [22]. Economic and physical accessibility for care are determinants of the ‘second delay’ [20]. Studies have found that distance to facilities is a clear barrier to women accessing health facilities [20,21], but in Addis Ababa, proximity to services does not appear to be a problem, as the median distance to a facility that provides surgical services is 5 kilometers, well below the national average of 45 kilometers [7]. Additionally, although the success of these efforts is uneven, the Ethiopian government has been working hard to eliminate financial barriers to care, through healthcare financing reforms introduced in 2005 [23,24]. The elimination of user fees and construction and repurposing of clinic and hospital infrastructure in Addis Ababa is contributing to reducing the “second delay” but, as has been found in other studies, supply side barriers such as an inadequate number of maternity beds are preventing women from accessing appropriate care [16]. Determinants of the ‘third delay’ are based on the quality of care available within facilities, both through facility readiness, in terms of infrastructure and supplies, and provider capacity [16,25].

Our mixed methods study on barriers to provision of high quality emergency obstetric care from the perspective of healthcare providers working in a referral network in Addis Ababa, Ethiopia provides additional evidence about the relationship between the three delays and identifies key barriers that need to be addressed to improve outcomes: lack of dedicated transportation and communication infrastructure, as well as overcrowding at the referral hospital, that undermines the referral system; lack of systematic pre-service and in-service training in BEmONC; and lack of supportive supervision. As illustrated in Figure 1 and described by providers, these health system weaknesses exacerbate delays in women reaching appropriate care (second delay) and receiving appropriate care (third delay). Respondents also reported that delays in access to and the provision of quality emergency obstetric care can undermine women’s confidence in the health system, and discourage them from seeking facility-based care (first delay). The three delays contribute to maternal deaths and poor maternal health outcomes [8].Our discussion focuses on proposals to overcome or mitigate the impact of the barriers identified by providers.

First, while the established referral protocol for the network is well-known to providers, it is not followed because of lack of dedicated transportation and communication infrastructure. Greater availability of command post ambulances and dedicated phone lines or cell phones are feasible means of overcoming these practical difficulties. Referral protocols could be modified to allow health centers to refer to an alternate hospital if their primary referral hospital does not have a bed. Furthermore, the idea of referring low-risk women from the hospital to a lower-level facility could provide an effective mechanism for alleviating overcrowding. All of these interventions could contribute to smoother functioning of the referral system and reduce the number of women who visit multiple hospitals while in labor, as well as reducing the probability of women giving birth in transit or at home after seeking facility-based care.

Second, training providers in emergency obstetric care is critical for reducing maternal mortality [26].We found that in this urban referral network pre-service training in BEmONC was inadequate or inexistent and in-service trainings were not well established. The combination of gaps in training and staff rotation results in many of the healthcare delivery sites in the referral network not having providers who have been systematically trained with updated knowledge about how to handle obstetric emergencies. Health center directors sought to maximize the benefit of scarce in-service trainings through peer-to-peer sharing but this informal system is probably less comprehensive and lower quality than formal in-service training, and the majority of providers were not satisfied with their in-service training opportunities. The informal training described by respondents does not result in certification, provides no clear career progression for providers, and undermines managers’ capacity to ensure standardized skill levels among specific cadres of healthcare workers. Dependence on project-specific training was identified as a significant challenge. Given that pregnancy and childbirth area leading cause of death of women of reproductive age in Ethiopia and reducing the maternal mortality ratio is a stated national goal, establishing a standardized curriculum and funding the systematic, regular offer of in-service training that privileges BEmONC and CEmONC in collaboration with external donors is essential. BEmONC training for providers working in health centers and supportive supervision which emphasizes appropriate referral could also reduce pressure on the referral hospital by increasing the proportion of low-risk births that are managed at the health center level.

Third, supportive supervision is a key component of quality improvement [9,27,28]. However, respondents’ descriptions of supervision indicated that supportive supervision was not the norm and that when it occurred, it was project specific, donor driven, and had not focused on obstetric emergencies. Respondents’ demand for supportive supervision indicates that implementing regular supportive supervision visits that address the functioning of the emergency obstetric referral network as a key area for action can contribute to improving quality of care. A first step is to establish guidelines for the provision of supportive supervision at the health center level. Ensuring that supportive supervision becomes standard procedure will demand a host of other activities, such as training providers in supportive supervision, changing the accountability structure and establishing reporting mechanisms.

Finally, respondents expressed frustration with supervision that does not result in provision of the medical equipment and infrastructure improvements that are necessary to provide emergency obstetric services. This finding makes it clear that improving communication and transportation infrastructure, training and supervision alone cannot overcome all barriers to providing quality care. There is a need to integrate supervision with health system planning and budgeting to meet urgent infrastructure needs.

As discussed, despite relative urban advantage in coverage of facility births and skilled-birth attendance in Addis Ababa, institutional capacity to provide emergency obstetric and newborn care is overburdened compared to the country as a whole. The population of Addis Ababa is expected to increase by over 60% between 2010 and 2025 [29]. Demands for emergency obstetric care will only increase due to demographic growth and urbanization. Projected population growth could place already strained maternal health systems in jeopardy. Actions to improve the timely delivery of emergency obstetric care are needed now, and will only become more important as demand for emergency obstetric services increases alongside population growth in Addis Ababa.

## Conclusions

This study identified barriers to the provision of timely and high quality obstetric emergency care in Addis Ababa, Ethiopia from the perspective of healthcare providers. The functioning of the referral network, training, and supervision were prioritized for evaluation because they are central to the FMOH’s priorities for improving maternal health, have been shown to improve the quality of maternal health services, can be implemented in the short-to-medium term, and maximize the use of existing health system resources. According to healthcare providers, investments in transportation and communication infrastructure, in-service training in BEmONC, and supportive supervision are priorities to support the functioning of the referral system and to improve the timely delivery of quality of emergency obstetric care in Addis Ababa, now and in the future.

## Additional files

Additional file 1:
**This is the quantitative baseline assessment tool we used to question all providers in the system on their training experience, capacity, knowledge and job satisfaction.**
Additional file 2:
**This is the structured interview guide, that was used to collect qualitative data from providers on their perceptions of training, the referral network, and barriers to providing quality emergency obstetric care.**

## Abbreviations

AARHB: Addis Ababa Regional Health Bureau
ACIPH: Addis Continental Institute of Public Health
ANC: Ante Natal Care
BEmONC: Basic Emergency Obstetric and Newborn Care
BSC: Bachelor of Science
CEmONC: Comprehensive emergency Obstetric and neonatal care
EDHS: Ethiopian Demographic and Health Survey
EmONC: Emergency Obstetric and Newborn Care
FMOH: Ethiopian Federal Ministry of Health
HIV: Human Immunodeficiency Virus
IECS: Institute for Clinical Effectiveness and Health Policy
IRB: Institutional Review Committee
SPMMC: Saint Paul Millennium Medical College
SPSS: Statistical Package for Social Science
MCH: Maternal and Child Health
MgSO_4_: Magnesium Sulfate
MHTF: Maternal Health Task Force
MMR: Maternal Mortality Ratio
NGO: Non-governmental Organization
PMTCT: Prevention of Mother to Child Transmission
PPH: Postpartum Hemorrhage
UNFPA: United Nations Population Fund
WHO: World Health Organization
