# Supplementary material for: Barriers to providing quality emergency obstetric care in Addis Ababa, Ethiopia: Healthcare providers’ perspectives on training, referrals and supervision, a mixed methods study
Source: BMC Pregnancy Childbirth. 2015 Mar 29;15:74. doi: 10.1186/s12884-015-0493-4 (PMC4407297; doi:10.1186/s12884-015-0493-4)
Supplement: Additional file 1: — This is the quantitative baseline assessment tool we used to question all providers in the system on their training experience, capacity, knowledge and job satisfaction. [file 12884_2015_493_MOESM1_ESM.pdf]

Health Facility Networking for Maternal Health Project

MHTF, SPMMC, IECS & ACIPH

Information Sheet and Informed Consent

The title of our project is: *"Baseline Assessment* **Survey to evaluate capacity, self confidence and knowledge of providers on handling obstetric emergencies".**

Good morning/afternoon. My name is (Name). We are collecting this data for the study conducted by Maternal Health Task Force (MHTF) at Harvard School of Public Health in collaboration with St. Paul's Millennium Medical College (SPMMC), Addis Continental Institute of Public Health (ACIPH), and the Institute for Clinical Effectiveness and Health Policy from Argentina (IECS).

The overall goal of this project is to decrease maternal morbidity and mortality through the use of innovative and scalable quality intervention package. The main purpose of this survey is to evaluate health professionals´ knowledge and level of confidence when handling obstetric emergencies. The information obtained from the study is intended to be used to improve the quality of the service provided to people in the community. Having this in mind your department/facility is one of those selected for this study.

This information will only be used for the purposes of this evaluation; it will be kept confidential at Addis Continental Institute of Public Health data management unit and it will only be made available to the study team. Further, your name and that of your institution will not be specifically mentioned or referred to in the report that we will produce at the end of the evaluation.

Your participation in this study is voluntary. You may refuse to answer any question, and you may choose to stop the interview at any time .If you agree to participate in this study, you will be asked to answer a questionnaire. The questionnaire will be administered to all health professionals that serve pregnant women in hospitals or health centers participants before and after the intervention is implemented. The information obtained before the intervention will be compared with results obtained after the intervention.

The information that you are going to provide us will be kept strictly confidential. It will not be transferred to any other third parties including your colleagues and boss. The data collection form will contain the name of your institution (Hospital/primary care health facility) but will not contain your name or any other information that could directly identify you. Your honest feedback will help us evaluate the effectiveness of our proposal. The questionnaire will ask you to do the following: describe your role as a health professional performing obstetric care and how confident you are in performing certain procedures at your institution. You will also be asked to provide some personal information such as sex, age, year of graduation and profession.

If you have any further questions, you can ask now. If you have questions later, you can contact Dr. Delayehu Bekele Tel no. 251922 743743.

The benefits of this study includes that what we learn from this study may benefit women, who in the future will receive obstetric care. Besides you will not be paid if you participate in this study or you will not have to pay if you decide to participate.

This survey will take us about 20-30 minutes to finish.

Participant's Statement

The reason as to why this study is conducted has been explained to me and I have agreed to take part. I have been given a chance to ask any questions I may have and my questions have been answered to my satisfaction. I understand that the information collected through my participation will be kept confidential /private. I understand that I may withdraw from this study at any time. My withdrawal from this study or my refusal to participate will in no way affect my working conditions at this centre or at any other centres. I agree to participate in this study voluntarily.

Participants name (print):____

____

Signature of Participant / (or mark of consent)   Date

Witness name (print): ____

Witness signature: _______________________________Date _______________________

Section I: Background Characteristics			
SN	Questions	Coding Categories	Skip
101	Health facility where the provider work	Hospital-1<br>Primary Healthcare Centre-2	
102	Name of Health Facility	____________________________________	
103	Code of Facility	[___|___]	
104	Date of interview	(dd/mm/yyyy): _____________________	
105	Time Interview Started	[___|___:___|___]	
106	Time Interview Ended	[___|___:___|___]	
107	Sex of provider	Male-1<br>Female-2	
108	Age of provider	[___|___]	
109	For how many years have you worked in the health care system?	[___|___]<br>If less than a year record "00"	
110	How long have you worked in this facility?	[___|___] Years<br><br>[___|___] Months<br><br>If less than a month record "00"	
111	Which section in the health facility are you currently working in?	Admission Room-1<br>Antenatal room/ward-2<br>Postnatal ward-3<br>Nursery-4<br>Other (Specify)_____________________-5	
112	For how many years have you worked in this section of the health facility ?	[___|___]<br>If less than a year record "00"	
113	What is your professional qualification?	Doctor-1<br>Health officer-2<br>Medical Intern -3<br>BSC Nurse/ Diploma nurse-4<br>/BSC Midwife/Diploma midwife -5<br><br>Other (Specify) _______________________-6	

Section II: Areas of In-Service training

For the following questions please circle one best response. Have you received in-service training in the following areas in a specified time period?

SN	Questions	Coding Categories							
		In the last one year				In the last three months			
		Yes	No	Don't know	Not applicable	Yes	No	Don't know	Not applicable
201	Comprehensive antenatal care/CANC*	1	2	8	9	1	2	8	9
202	Assess progress of labour (use of the partograph and assessment of cervical conditions)	1	2	8	9	1	2	8	9
203	Management of normal labour and delivery	1	2	8	9	1	2	8	9
204	Active management of third stage labor: i.e. Administer uterotonic <u>and</u> uterine massage <u>and</u> controlled cord traction	1	2	8	9	1	2	8	9
205	Administer intravenous fluids	1	2	8	9	1	2	8	9
206	Targeted Postpartum care for the mother	1	2	8	9	1	2	8	9
207	Recognize antepartum haemorrhage	1	2	8	9	1	2	8	9
208	Recognize postpartum hemorrhage	1	2	8	9	1	2	8	9
209	Perform aspiration for retained products	1	2	8	9	1	2	8	9
210	Perform curettage for retained products	1	2	8	9	1	2	8	9
211	Administer parenteral (intravenous or injection) uterotonics for post-partum haemorrhage	1	2	8	9	1	2	8	9
212	Repair vaginal or cervical tears	1	2	8	9	1	2	8	9
213	Administer blood transfusion	1	2	8	9	1	2	8	9
214	Recognize preeclampsia	1	2	8	9	1	2	8	9
215	Recognize eclampsia	1	2	8	9	1	2	8	9
216	Administering of magnesium sulphate for the management of eclampsia/pre-eclampsia	1	2	8	9	1	2	8	9
217	Management of eclampsia/ high blood pressure in pregnancy labor and after delivery	1	2	8	9	1	2	8	9
218	Perform manual removal of placenta	1	2	8	9	1	2	8	9
219	Perform a forceps delivery	1	2	8	9	1	2	8	9
220	Perform a vacuum delivery	1	2	8	9	1	2	8	9
221	Make and repair episiotomy	1	2	8	9	1	2	8	9
222	Perform a caesarean section	1	2	8	9	1	2	8	9
223	Perform uterine artery ligation	1	2	8	9	1	2	8	9
224	Repair ruptured uterus	1	2	8	9	1	2	8	9
225	Perform an hysterectomy	1	2	8	9	1	2	8	9
226	Care of the newborn (Essential newborn care)	1	2	8	9	1	2	8	9
227	Neonatal resuscitation	1	2	8	9	1	2	8	9
228	Adult resuscitation	1	2	8	9	1	2	8	9
229	Other Specify………………………………………………………………………………………………………	1	2	8	9	1	2	8	9

---

* Comprehensive antenatal care includes the following components: (1) screening, monitoring, and testing for early identification and management of medical problems or complications; (2) ongoing assessment and mediation of risk factors (such as tobacco and alcohol use or domestic violence); (3) nutrition assessment and counseling; (4) health education, information, and counseling about pregnancy, labor and delivery, and baby care; and (5) assessment and care for psychosocial needs (such as stress reduction).

Section III: Provider Self efficacy, Skill and Knowledge

301. Have you performed the following skills in the last three months?

SN	Questions	Coding Categories					
		Yes	No	Not applicable	302. Do you feel comfortable performing this skill?		
					Yes	No	Not applicable
a.	Administer intravenous fluids	1	2	9	1	2	9
b.	Perform active management of third stage labor: i.e. Administer uterotonic and uterine massage and controlled cord traction	1	2	9	1	2	9
c.	Perform vacuum aspiration for retained products	1	2	9	1	2	9
d.	Perform curettage for retained products	1	2	9	1	2	9
e.	Administer parenteral (intravenous or injection) uterotonics for post-partum haemorrhage	1	2	9	1	2	9
f.	Administer blood transfusion	1	2	9	1	2	9
g.	Administer magnesium sulphate for the management of eclampsia/pre-eclampsia	1	2	9	1	2	9
h.	Administer other anticonvulsants for the management of eclampsia/preeclampsia	1	2	9	1	2	9
i.	Perform manual removal of placenta	1	2	9	1	2	9
**303. During *<u>the last three months</u>* have you made your own decisions or took direct orders from another health worker, or acting according to Standard Operating Procedures to perform the following activities:**		If answer to question 301 was "YES"			If answer to question 301 was "NO"	If answer to question 301 was "Not Applicable"	
		Independent Decision	Direct Orders	Standard Operating Procedures			
a.	Administer intravenous fluids	3	4	5	2	9	
b.	Perform active management of third stage labor: i.e. Administer uterotonic and uterine massage and controlled cord traction	3	4	5	2	9	
c.	Perform vacuum aspiration for retained products	3	4	5	2	9	
d.	Perform curettage for retained products	3	4	5	2	9	
e.	Administer parenteral (intravenous or injection) uterotonics for post-partum hemorrhage	3	4	5	2	9	
f.	Administer blood transfusion	3	4	5	2	9	
g.	Administer magnesium sulphate for the management of eclampsia/pre-eclampsia	3	4	5	2	9	
h.	Administer other anticonvulsants for the management	3	4	5	2	9	

	of eclampsia/preeclampsia					
i.	Perform manual removal of placenta	3	4	5	2	9

	**Level of knowledge**							
**304. How would you define your current level of knowledge to perform the following procedures**		**None**	**Poor**	**Adequate**	**Good**	**Excellent**	**Not Applicable**	
a.	Emergency Preeclampsia diagnosis	1	2	3	4	5	9	
b.	Emergency Preeclampsia treatment	1	2	3	4	5	9	
c.	Eclampsia diagnosis	1	2	3	4	5	9	
d.	Eclampsia treatment	1	2	3	4	5	9	
e.	Use of magnesium sulfate	1	2	3	4	5	9	
f.	Postpartum hemorrhage diagnosis	1	2	3	4	5	9	
g.	Postpartum hemorrhage treatment	1	2	3	4	5	9	
h.	Urgent c/section	1	2	3	4	5	9	
i.	Urgent hysterectomy	1	2	3	4	5	9	
j.	Maternal resuscitation	1	2	3	4	5	9	
	**Level of Confidence**							
**305. How do you rate your level of confidence on the following areas?**		**None**	**Poor**	**Adequate**	**Good**	**Excellent**	**Not Applicable**	
a.	In interacting with the rest of the team during an obstetric emergency	1	2	3	4	5	9	
b.	That my decisions are respected by the other members of the team	1	2	3	4	5	9	
c.	In interacting with other units/providers during obstetric emergencies (e.g. anesthesiologist, midwifes, obstetricians, lab.)	1	2	3	4	5	9	
**306. On a scale of 1 to 5 how would you rate your level of confidence to clinically manage the following situations?**								
a.	Postpartum hemorrhage	1	2	3	4	5	9	
b.	Maternal resuscitation	1	2	3	4	5	9	
c.	Preeclampsia	1	2	3	4	5	9	
d.	Eclampsia	1	2	3	4	5	9	
307. On a scale of 1 to 5 how would you rate your level of confidence to assist in the clinical management the following situations?								

a.	Postpartum hemorrhage	1	2	3	4	5	9	
b.	Maternal resuscitation	1	2	3	4	5	9	
c.	Preeclampsia	1	2	3	4	5	9	
d.	Eclampsia	1	2	3	4	5	9	

Section IV: LEVEL OF JOB SATISFACTION				
**401. On the scale of 1 to 4 how would you rate your job satisfaction**	Very satisfied	Satisfied	Indifference	Not satisfied at all
The freedom I have to make important patient care and make an independent decision	4	3	2	1
The collaboration or team work or practice between different cadres of health workers	4	3	2	1
Availability of supplies and equipment to perform duties	4	3	2	1
Accomplishment of many worthwhile things in this job	4	3	2	1
Training opportunities given by the health facility for providers	4	3	2	1
The method utilized to decide who gets promoted in the health facility	4	3	2	1
The method of supervision utilized in the health facility	4	3	2	1

Note for the data collector: Don´t forget to complete question 106 ("Time Interview Ended")

Thank you for your time!
