# Supplementary material for: Barriers to providing quality emergency obstetric care in Addis Ababa, Ethiopia: Healthcare providers’ perspectives on training, referrals and supervision, a mixed methods study
Source: BMC Pregnancy Childbirth. 2015 Mar 29;15:74. doi: 10.1186/s12884-015-0493-4 (PMC4407297; doi:10.1186/s12884-015-0493-4)
Supplement: Additional file 2: — This is the structured interview guide, that was used to collect qualitative data from providers on their perceptions of training, the referral network, and barriers to providing quality emergency obstetric care. [file 12884_2015_493_MOESM2_ESM.pdf]

Evaluating capacity enhancement package in a network of health facilities

Information Sheet and Informed Consent

Good morning/afternoon. My name is (**Name**). We are collecting this data for a study conducted by Maternal Health Task Force (MHTF) at Harvard School of Public Health in collaboration with St. Paul's Millennium Medical College (SPMMC), Addis Continental Institute of Public Health (ACIPH), and the Institute for Clinical Effectiveness and Health Policy from Argentina (IECS).The main purpose of this study is to develop an intervention with the goal to significantly improve the quality care services for women during labor and delivery at the health facilities. Specifically, we will try to identify factors and challenges that might hinder quality Improvement efforts in health facilities. In order to do so, we would like to hear from health workers such as yourself about the availability of resources and quality of emergency obstetric health services for women in this area.

I will give you information and invite you to participate in this research by taking part in an in depth interview during the formative phase of the research. This interview will take about 45 minutes. This phase is in preparation for the implementation of an intervention package to improve the quality of maternal health services. If you decide to participate, you will receive a copy of this consent form.

You are being invited to take part in this research because we feel that your experience as a health care worker in Addis Ababa can contribute much to our understanding and knowledge of local health practices.Your thoughts and opinions are valuable to us, so please feel free to interrupt us anytime during the interview if you want to raise an issue or seek clarification.You may also skip any questions which you don't want to answer during the interview so that the interviewer will move to the next question. I will be leading the discussion and a colleague of mine will be present to take notes. In addition, because it might be difficult to jot down everything while we discuss, we might tape record our conversation to be sure that we don't miss anything but no-one will be identified by name on the tape only unique codes will be given to the tapes for identification. The tape with the information recorded will be kept confidential at Addis Continental Institute of Public health data management unit where no one will have access to the information documented during your interview. except the research team. The tapes will be destroyed after four weeks of data collection.

This information will only be used for the purposes of this evaluation; it will be kept confidential and it and will not be transferred to your colleagues or seniors. The study team believes that participating in this study does not carry any risks and you will not suffer from any harm or injury because of your participation. Further, your name and that of your institution will not be specifically mentioned or referred to in the report that we will produce at the end of the evaluation.

Do you have any questions for me at this time?

(***Respondent asks the questions and the note taker writes them down***)

Issues to Cover

Our discussion will cover the following topic/issues:

We would like to ask your insights about the capacity of facilities in terms of supplies, equipment and staff available to provide a number of life-saving essential obstetric functions. These life-saving functions are those used to treat the most common or major obstetric complications including hemorrhage (antepartum, intrapartum, or postpartum);prolonged/obstructed labor; pre-eclampsia/eclampsia; and ruptured uterus. In addition, during the interview we will also discuss the quality of care provided.

Confidentiality of your information: A data collection form will contain the name of the health facility you are working in but it will not contain your name or any other information that could directly identify you. Your name and that of your institution will not be specifically mentioned or referred to in the report that we will produce at the end of the evaluation. The data collection forms which contain this information will be kept confidentially at ACIPH data management unit where except the research team no one will access them.

Voluntary participation: The choice that you make about whether to participate in this study will have no bearing on your job or on any work-related evaluations or reports. You may change your mind later and stop participating during the interview even if you sign this Informed Consent form.You may stop participating in the interview at any time that you wish without your job being affected. I will give you an opportunity at the end of the interview to review your remarks, and you can ask to modify or remove portions of those, if you do not agree with my notes or if I did not understand you correctly.

Who to call for information: If you have any questions, please do not hesitate to ask me at this time. If you have questions later, you can contact Dr.Delayehu Bekele(tel no. 251922 743743).

This proposal has been reviewed and approved by ACIPH-IRB, which is a committee whose task it is to make sure that research participants are protected from harm. If you wish to find about more about the IRB, contact Professor Yemane Berhane, +251911219785, Addis Ababa.

What are the benefits of this research? What we learn from this study may benefit future women and their children who receive health facility medical care during delivery. We hope that the results of this research will improve quality of care during delivery and help save lives.

Research costs: You will not be paid to participate in this study. If you decide to participate, there is no cost.

Do you have any questions for me at this time?

(***Respondent asks the questions and the note taker writes them down***)

Participant's Statement/Certificate of Consent

The reason why this study is being conducted has been explained to me and I have agreed to take part. I have been given a chance to ask any questions I may have and my questions have been answered to my satisfaction. I understand that the information collected through my participation will be kept confidential /private. I understand that I may withdraw from this study at any time. My withdrawal from this study or my refusal to participate will in no way affect my working conditions at this centre or at any other centres.

I have read the foregoing information, or it has been read to me. I have had the opportunity to ask questions about it and any questions I have been asked have been answered to my satisfaction. I consent voluntarily to be a participant in this study.

Participants name (print):____

____ _________________

Signature of Participant / (or mark of consent) Date

Witness name (print): ____

Witness signature: _________________________________Date ________________________

Capacity Enhancement in the EmONC services to improve the quality of care of women having obstetrical emergencies in Ethiopia

SECTION I –Background Information

Age	
Sex	Male-1<br>Female-2
Code of the Interviewee	
Qualification/Degree	
Position	
Duration of service at current health center	Years [___|___]<br>Months [___|___]
Duration in the current position	Years [___|___]<br>Months [___|___]
Type of health institution	Health Center-1<br>Hospital-2
Date of interview	
Time interview began	
Time interview ended	
Interviewer Name	
Interviewer additional observations and remarks	

SECTION II –Interview Guide for Providers

No.	**Objective: Capacity of Providers**
	Q1: Are the staff in the facility that you are working in provided with emergency obstetric skill trainings?<br>A. If yes, what are the specific areas on which trainings are given?<br>B. Were there any criteria for the involvement of providers in the training? How many of the staff members were involved in each specific area of training? When?<br>C. On a scale of 1 to 5, how do you rate the capacity of the providers in your facility to handle emergency obstetric cases?**1-None, 2-Poor, 3- Adequate, 4-poor, 5-Excellent.** Why?<br>D. Do you think more training is needed to enhance the capacity of providers in handling emergency obstetric cases? If yes, what specific areas of trainings are required? Why?
	**Objective: Referral and Networking**
	Q1: How are the referral and the networking systems functioning both within your facility and between your health facility and other health facilities like hospitals?<br>A. Are there review meetings conducted among MNCH staff? If yes, how frequently are they conducted? What are the major issues usually discussed in these meetings?<br>B. What, if any, changes in referral practices have come out of past review meetings?<br>C. Are there any issues which were not addressed in the past review meetings that need an improvement? If yes can you mention some of them?<br>D. Is there an intra-facility referral and communication system in this health facility? If yes, can you please explain how it is performing? Please mention any problems with the referral hospital responding to referral requests.<br>E. Are mothers with obstetric complications referred to hospitals? Which hospital does your facility usually refer to? Please explain the mechanism of referring women with obstetric complications to other health facilities/hospitals. How is it determined which women get referred? Who makes the referral? Does anyone from the HC go with the woman to the referral hospital? How long does the referral process take?<br>F. What, if any, mechanism do you use to trace referral cases? Can you please explain? And also can you explain the back referral mechanism that you are using if you have one?<br>G. What are the problems with having referrals to the hospital?<br>H. Please describe the process ofdocumenting referrals, communication (Both intra and inter facility) and thereporting system in the facility you are currently working in. What, if any, documentation is used?
	**Objective: Group supportive supervision and exchange of knowledge**
	Q1: What, if any, kinds of support do you get from other health facilities/hospitals or health bureaus?<br>A. In which part does the support mainly focus? (This could be administration, a specific area of maternal care, etc.) Is it on a frequent basis?<br>B. What type of support do you believe benefits you the most?<br>C. Is there anything that you would like to comment on the support you received? Please speakin terms of relevance to the problems and timelinessof the support that was received.<br>D. Have you ever received supportive supervision* from health facilities/hospitals or health bureaus? Can you please tell me about your experiences in being supervised; both the negative and positive aspects?<br>E. Have you ever conducted or participated in a knowledge exchange program between the

	health facility in which you work and St. Paul, and or between your health facility and another health facility in St. Paul's catchment area?If yes, where? What was the main topic on which knowledge was exchanged?<br><br>*If the interviewee needs clarification, provide the following definition for supportive supervision: supervision which includes observation, discussion, support in guidance of the maternal health services provided, standard of care practices, equipment supplies and infrastructure from regional health bureau/Addis Ababa regional health bureau, referral hospital management etc
